# Transcriptome of the *Aedes aegypti* Mosquito in Response to Human Complement Proteins

**DOI:** 10.3390/ijms21186584

**Published:** 2020-09-09

**Authors:** Gloria I. Giraldo-Calderón, Arley Calle-Tobón, Paula Rozo-López, Tonya M. Colpitts, Yoonseong Park, Guillermo L. Rua-Uribe, Berlin Londono-Renteria

**Affiliations:** 1 VectorBase, Department of Biological Sciences, University of Notre Dame, Notre Dame, IN 46556, USA; ggiraldo@nd.edu; 2 Departamento de Ciencias Biológicasy, Universidad Icesi, Calle 18 No. 122–135, 760020 Cali, Colombia; 3 Departamento de Ciencias Básicas Médicas, Universidad Icesi, Calle 18 No. 122–135, 760020 Cali, Colombia; 4 Department of Entomology, Kansas State University, Manhattan, KS 66506, USA; arley@ksu.edu (A.C.-T.); paularozo@ksu.edu (P.R.-L.); ypark@ksu.edu (Y.P.); 5 Grupo Entomología Médica, Universidad de Antioquia, 050001 Medellín, Colombia; guillermo.rua@udea.edu.co; 6 Department of Microbiology & National Emerging Infectious Diseases Laboratories, Boston University School of Medicine; Boston, MA 02118, USA; tmcol@bu.edu

**Keywords:** human complement, *Aedes aegypti*, blood-meal, hC3, hC5a

## Abstract

*Aedes aegypti* is the primary mosquito vector of several human arboviruses, including the dengue virus (DENV). Vector control is the principal intervention to decrease the transmission of these viruses. The characterization of molecules involved in the mosquito physiological responses to blood-feeding may help identify novel targets useful in designing effective control strategies. In this study, we evaluated the in vivo effect of feeding adult female mosquitoes with human red blood cells reconstituted with either heat-inactivated (IB) or normal plasma (NB). The RNA-seq based transcript expression of IB and NB mosquitoes was compared against sugar-fed (SF) mosquitoes. In in vitro experiments, we treated Aag2 cells with a recombinant version of complement proteins (hC3 or hC5a) and compared transcript expression to untreated control cells after 24 h. The transcript expression analysis revealed that human complement proteins modulate approximately 2300 transcripts involved in multiple biological functions, including immunity. We also found 161 upregulated and 168 downregulated transcripts differentially expressed when human complement protein C3 (hC3) and human complement protein C5a (hC5a) treated cells were compared to the control untreated cells. We conclude that active human complement induces significant changes to the transcriptome of *Ae. aegypti* mosquitoes, which may influence the physiology of these arthropods.

## 1. Introduction

*Aedes* mosquitoes, especially *Ae. aegypti* and *Aedes albopictus* are responsible for major arboviral epidemics of yellow fever (YFV), dengue (DENV), chikungunya (CHIKV), and Zika (ZIKV) around the world [1,2]. Mosquitoes acquire these viruses from a vertebrate host following the ingestion of an infected blood meal [3]. Once inside the mosquito, viruses must overcome several barriers before they can be transmitted back to a vertebrate host [4]. The first barrier is the midgut, where the pathogen would need to reach permissive cells before it is attacked by the mosquito digestive enzymes and active factors contained in the blood meal that can cause lysis of the virions [4,5]. Surviving pathogens must then penetrate the midgut tissue, replicate, disseminate, and reach the salivary glands, representing the second barrier, and the last door to exit the vector through saliva during their next feeding on a susceptible vertebrate host [6]. Therefore, understanding the mechanisms that mediate the colonization of the vector tissues by the virus is vital to determining vector competence and transmission dynamics, and finding novel mechanisms to prevent disease transmission.

During the blood-feeding on a human host, plasma factors remain active for several hours inside the mosquito midgut, influencing mosquito physiology [7]. Among those factors, activation of the complement system may damage the insect tissue and reduce the population of microorganisms present in it, representing an important limiting factor on pathogen survival during their first hours inside the arthropod [8,9,10]. The human complement is a system that comprises more than 30 soluble proteins and membrane-bound receptors, mainly found in their inactive forms [11,12]. Upon activation, they generate products that opsonize (or coat) microorganisms and promote their phagocytosis by immune cells, among several other mechanisms to directly eliminate the pathogen, such as causing lysis through the formation of the membrane attack complex or MAC [13]. The MAC is an effective mechanism to destroy a wide variety of pathogens, including parasites, bacteria, and enveloped viruses [12,14]. However, in the case of non-enveloped viruses opsonization and MAC formation on virus-infected cells have been described [14].

The human complement system can be divided into three main pathways: classical (activated by antibodies), alternative (constitutively activated by autocatalysis of C3 protein), and the lectin pathways (characterized by serine protease enzymatic activity). The human complement proteins C3 (hC3) and C5 (hC5) are among the most important factors. For instance, hC3 is the central player as the three conventional complement pathways converge [15] and the most abundant complement protein in plasma [16]. hC3 convertases cleave this protein into hC3a and hC3b [16,17]. Furthermore, hC5a (a product of hC5 cleavage) works as a chemoattractant for neutrophils and macrophages, which can also enhance phagocytosis (through opsonization of virions and infected cells), stimulating the release of granules and activating coagulation [18]. hC3 and hC5 factors are highly active in the mosquito midgut up to 3 h after feeding [10], suggesting that they may still exert their antiviral activity during that period.

Complement activation during DENV infection in humans is a well-documented event, and increased levels of complement proteins are found in DENV-infected patients compared to uninfected individuals [19,20,21]. Interestingly, the vast majority of human infections by DENV are asymptomatic (~70%) [22], and these individuals present lower systemic complement activation [23]. In contrast, higher activation of the complement system and an excess of production of hC5a have been observed in severe dengue [24,25,26]. We hypothesized that the complement activation in human blood may be detrimental to DENV infection in the mosquito midgut and that viremic asymptomatic individuals may still be infectious to mosquitoes. Moreover, asymptomatic individuals may also contribute significantly to virus transmission due to their uninterrupted lifestyle [27] compared to severe DENV cases that are mostly confined to the hospital. We speculate that lower complement activation in asymptomatic cases may be an important factor in modulating infection and transmission in the mosquito vector.

Previous studies suggested that decreasing DENV replication in the mosquito may be related to expression changes in *Ae. aegypti* macroglobulin complement-related proteins (AaMCR) and antimicrobial peptides (AMP) [28]; and that hC5a may interact with midgut uncharacterized proteins increasing the AMP, defensin A [8]. However, the exact mechanisms leading to the observed complement-dependent decrease in infection remain unknown. The objective of this study was to evaluate the effect of human complement on the transcriptome of *Ae. aegypti* mosquitoes after blood-feeding (in vivo) or after direct exposure of the Aag2 cell line (in vitro). Our current study suggests that human complement, hC3 and hC5a proteins induce changes in the transcriptome of *Ae. aegypti*, both in vivo and in vitro, and regulate a significant number of transcripts, including AMPs, related to immune responses. We conclude that the changes in the transcriptome of *Ae. aegypti* mosquitoes could be used to find novel ways of decreasing mosquito survival and inhibit virus transmission. Our rationale to focus on mosquito proteins that are activated against human complement is that these proteins could represent alternative candidates for control interventions, such as transmission-blocking vaccines (TBVs), in contrast to using viral proteins as targets because these may be more prone to mutation due to the immunological pressure exerted by both vertebrate and invertebrate hosts.

## 2. Results

### 2.1. Sequencing and Read Mapping 

To understand the molecular interactions between human complement and *Ae. aegypti* (Rockefeller strain), RNA-sequencing was used to explore the changes in the mosquito transcriptome. More than 135 million (135,411,293), Illumina 50 bp single-end directional reads (strand-specific) were sequenced across all samples (Table S1). Each in vitro test was represented by three libraries with an average of 9.13 million reads per library. The in vivo tests were represented by two libraries with an average of 8.87 million reads per library. The results obtained from the mapping of processed reads (131,210,742) against *Ae. aegypti* Liverpool Aedes Genome Working Group (LVP_AGWG) AaegL5.2 showed a higher percentage of uniquely mapped reads using the Spliced Transcripts Alignment to a Reference (STAR), showing between 6.71 to 7.72% more mapped reads than using Hisat2. Thus, these were used for expression analysis.

### 2.2. Transcript Differential Expression in NB and IB Fed Mosquitoes (In Vivo Experiments)

RNA-sequencing was conducted to explore the transcriptome changes in the *Ae. aegypti* abdominal region in response to oral ingestion of human red blood cells reconstituted with either autologous heat-inactivated plasma (IB) or normal plasma (NB) at 3 h post-feeding. RNA-seq analyses showed that 70.1% (19,866 out of 28,353) of annotated protein-coding transcripts were expressed in the mosquitos. A total of 3369 out of 28,353 of annotated protein-coding transcripts (equivalent to 11.9% of transcripts) were found differentially expressed (Fold change (FC) >4, *p* < 0.05) when comparing NB and sugar-fed (SF) control females. In this comparison, we found 1452 up- and 1917 downregulated transcripts. 

Furthermore, 3270 out of 28,353 transcripts were found differentially expressed in SF when compared against IB (1496 upregulated and 1774 downregulated) (Table S2). The comparison of NB vs. IB showed 1065 transcripts up- and 1285 downregulated differentially expressed in NB (Table S2). Further analysis of transcripts found differentially expressed in both NB and IB when compared to sugar-fed mosquitoes revealed 733 upregulated and 795 downregulated transcripts found in common.

**Figure 1:**
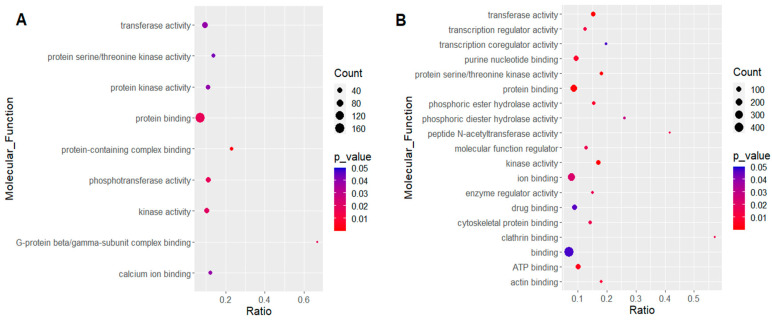
Gene Set Enrichment Analysis (GSEA) of molecular functions for differentially expressed transcripts after feeding with normal plasma (NB) and compared to those fed with heat-inactivated plasma (IB). (**A**) Upregulated transcripts. (**B**) Downregulated transcripts.

Gene Set Enrichment Analysis (GSEA) of molecular functions (MF) based on Gene Ontology (GO) was conducted on the 8.3% differentially expressed transcripts in response to NB vs. IB (Figure 1). Differentially expressed transcripts were highly clustered in several molecular functions, such as protein binding and kinase activity for upregulation. Some of the transcripts found upregulated in the NB fed group were associated with functions as ubiquitin ligase complex (AAEL010793-RB, AAEL010147-RJ, AAEL007694-RE, AAEL004875-RB, AAEL003466-RG, AAEL013530-RB, AAEL007187-RB); proteins involved in oxidative stress and heme-binding (AAEL000342-RF (peroxidasin), AAEL023523-RD, AAEL007563-RG (Dual Oxidase: Peroxidase and NADPH-Oxidase domains), AAEL003762-RC (Protoporphyrinogen IX oxidase), AAEL005108-RA (manganese-iron (Mn-Fe) superoxide dismutase), AAEL005478-RN (flavohemoprotein B5/b5r), AAEL012580-RA (3-hydroxyisobutyrate dehydrogenase), AAEL010580-RA (3-hydroxyisobutyrate dehydrogenase, putative), AAEL003762-RC (Protoporphyrinogen IX oxidase), AAEL005400-RD (2-hydroxyacid dehydrogenase), AAEL010158-RB (cytochrome P450), AAEL004457-RD (cytochrome c), and AAEL010393-RA (ferritin subunit, putative).

In contrast, downregulated transcripts in the NB compared to the IB were mainly associated with functions related to carbohydrate metabolism (AAEL002781-RG (galactokinase), AAEL009387-RA (hexokinase)); transport and storage of lipids (AAEL006982-RG (lipase), AAEL006667-RF (phosphatidyltransferase), AAEL007996-RA (centaurin alpha), AAEL003402-RI (sphingomyelin phosphodiesterase)); cell signaling (AAEL008847-RF (wingless), AAEL004846-RD (Protein lin-7 homolog), AAEL008679-RC (Alpha-tubulin N-acetyltransferase)); regulation of nitrogenous compounds metabolism (AAEL009099-RD (uridine cytidine kinase), AAEL011698-RB (mRNA (guanine-7-) methyltransferase), AAEL013653-RB (tata-box binding protein), AAEL000185-RD (eukaryotic translation initiation factor), AAEL004132-RE (Mediator of RNA polymerase II transcription subunit 31), AAEL004385-RB (UGA suppressor tRNA-associated antigenic protein)); as well as ~73 transcripts involved in transcription regulation.

### 2.3. Transcript Differential Expression in Aag2 Cells upon Treatment with hC3 and hC5a (In Vitro Experiments)

RNA-seq analyses showed that 54.4% (15,422/28,353 AaegL5.2) of all annotated *Ae. aegypti* protein-coding transcripts were expressed in hC3-treated, hC5a-treated, and control untreated Aag2 cells. A total of 2.6% (749/28,353) transcripts were identified with differential accumulation when comparing hC3-treated cells vs. control cells, with 324 and 425 transcripts up- and downregulated, respectively (Table S2). The upregulated transcripts were enriched in phosphotransferase and oxidoreductase activities (AAEL008841-RC, AAEL008574-RB, AAEL003762-RD). While the downregulated showed a significant increase in the kinase activity functions and binding proteins involved in the regulation of cellular processes (AAEL009041-RG, AAEL006510-RC, AAEL019921-RB, AAEL010926-RD) and the ubiquitin ligase complex (AAEL013530-RB, AAEL003466-RH, AAEL007694-RD, AAEL004875-RA, AAEL004697-RA).

The comparison between hC5a-treated cells and the untreated control cells revealed that 3.5% (981/28,353) of the transcripts had significant differential expression (504 upregulated and 477 downregulated) (Table S2). The upregulated transcripts were enriched in ion binding, phosphotransferase activity, and nucleotide-binding functions. In contrast, the downregulated transcripts were associated with functions such as actin-binding, cytoskeletal protein binding, guanosine-5’-triphosphate (GTP)ase regulator activity, and regulation of catalytic activity. 

**Figure 2:**
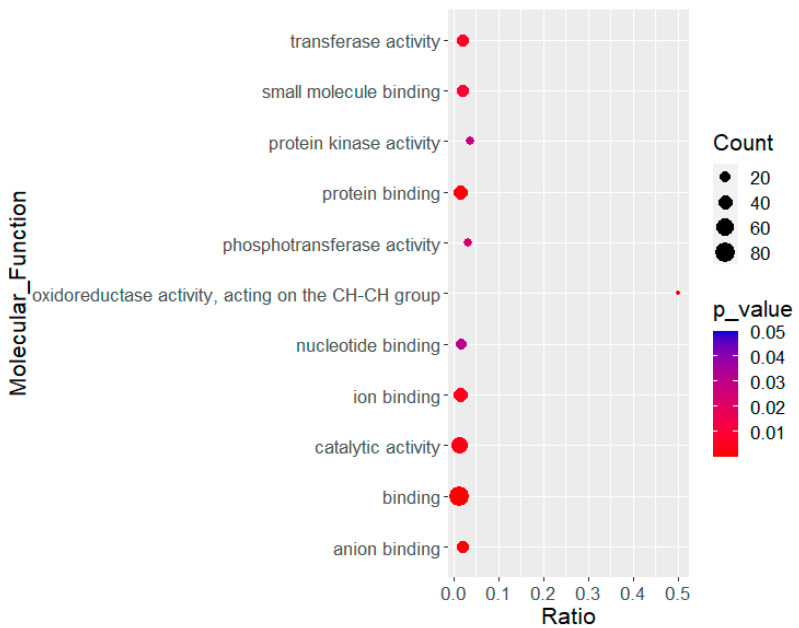
Gene Set Enrichment Analysis (GSEA) of molecular functions for the commonly upregulated transcripts in Aag2 cells treated with hC3 and hC5a and compared against untreated control cells.

A total of 1.2% (329/28,353) differentially expressed transcripts, 161 up- and 168 downregulated, were in common between hC3 and hC5a treatments. The upregulated transcripts presented enrichment in multiple molecular functions like binding, regulation, and oxidoreductase activity. The GSEA of transcript differentially expressed in cells treated with either hC3 or hC5a compared to untreated cells is shown in Figure 2.

### 2.4. Changes in the Immune Response Transcripts (IRTs) to Complement in the In Vivo and In Vitro Experiments

**Figure 3:**
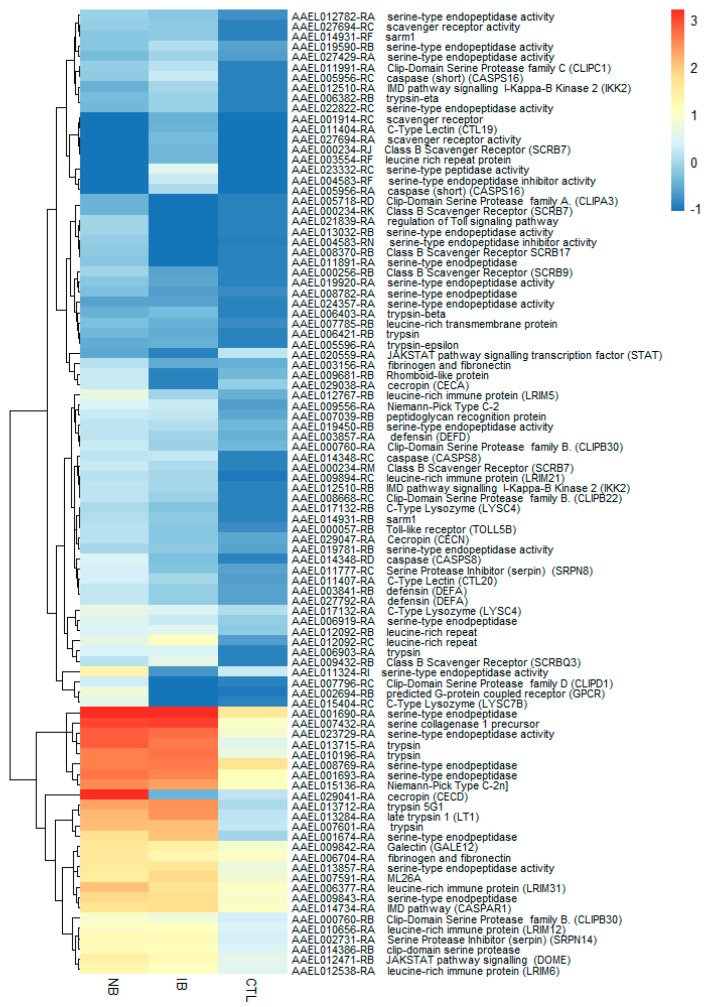
Heatmap showing the expression profile of 94 immune-related transcripts (IRT) transcripts found upregulated.

**Figure 4:**
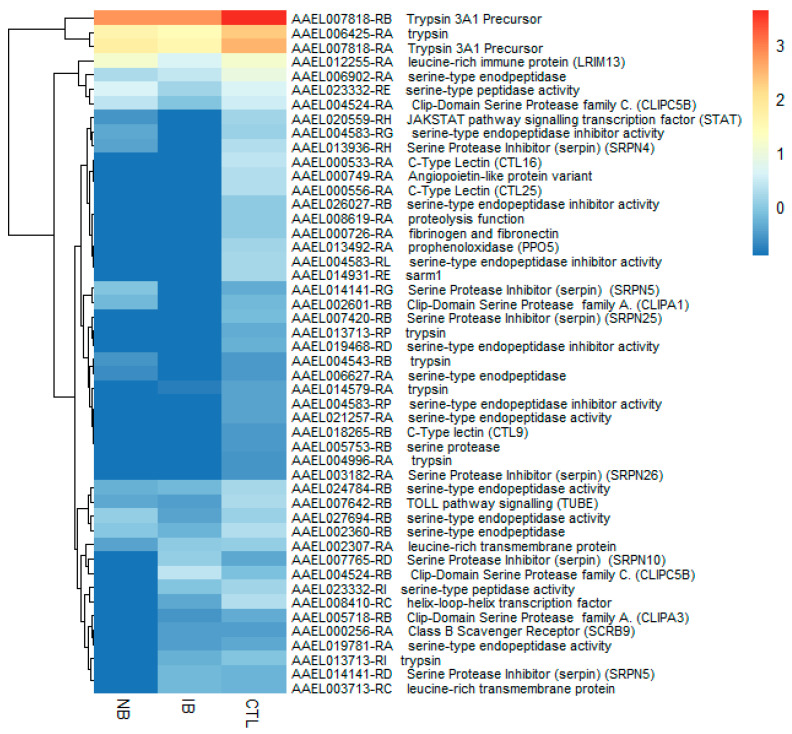
Heatmap showing the expression profile of 48 IRT found as downregulated in the in vivo treatments. Normal blood (NB). Heat-inactivated blood (IB). Sugar meal (Ctl).

Within the set of transcripts that were differentially expressed in *Ae. aegypti* after in vivo experiments, 142 transcripts (94 up- and 48 downregulated) were involved in immune responses and other processes, such as apoptosis and autophagy; these represent ~16% (142/883) of the total IRTs (Figure 3, Figure 4 and Table S3). Seventy-seven transcripts were upregulated after feeding with NB, 52 were found upregulated after feeding with IB, and 35 were common to both treatments. The top nine most abundant transcripts, with a fold change from 2.15 to 8.3 (Log2FC), encoded multiple protein families like serine-type endopeptidases Clip-Domain Serine Protease family C, family D, family E (AAEL000760, AAEL005718, AAEL007796, AAEL008668); Class B Scavenger Receptors (AAEL000234, AAEL000256, AAEL008370, AAEL009432); C-Type Lectin (AAEL011404, AAEL011407); Cecropins (AAEL029038, AAEL029041, AAEL029047); Defensins (AAEL003841, AAEL003857); The immune deficiency (IMD) pathway signaling (AAEL012510, AAEL014734, AAEL015136); and one G-protein coupled receptor (GPCR) (AAEL002694).

Regarding the downregulated transcripts associated with IRTs, 48 transcripts were found (Table S3), of which 19 transcripts were common to both NB and IB fed mosquitoes. The downregulated transcripts families involved principally fibrinogen related proteins (AAEL000726-RA, AAEL000749-RA); C-Type Lectin (AAEL000533-RA, AAEL000556-RA, AAEL018265-RB); serine-type endopeptidase inhibitor activity (AAEL004583-RG, AAEL019468-RD, AAEL026027-RB); trypsin (AAEL004543-RB, AAEL004996-RA, AAEL006425-RA, AAEL007818-RB, AAEL014579-RA, AAEL007818-RA, AAEL013713-RP); and Serine Protease Inhibitor (Serpin) (AAEL007420-RB, AAEL013936-RH, AAEL014141-RG). Interestingly, two transcripts downregulated in IB but upregulated in NB were AAEL009681-RB (Rhomboid-like protein) with a 5.7-fold increase and AAEL029038-RA (Cecropin) with a 10.2-fold increase.

In the in vitro experiments, the Aag2 cells treated with hC3 and hC5a also showed a change in the expression of IRTs (Table S3). The cells treated with hC3 compared to control cells presented a change in the expression of 13 transcripts (6 up- and 7 downregulated), including the upregulation of caspase (AAEL014348-RF), Class B Scavenger Receptors (i.e., AAEL000234-RL, AAEL000256-RC), serine protease (AAEL002301-RB) and Sarm1 (AAEL014931-RD) transcripts. We observed a decrease in trypsins (AAEL007818-RA, AAEL013713-RO) and Serine Protease Inhibitors (serpins) (AAEL007765-RD, AAEL010769-RC). Moreover, comparing Aag2 cells treated with hC5a and control cells, 24 transcripts with differential expression were found (12 up- and 12 downregulated in hC5a-treated cells). Specifically, we observed an increase in the expression of serine-type peptidases (AAEL013032-RC, AAEL023332-RC), serine-proteases (AAEL002301-RB, AAEL002601-RB), vesicular mannose-binding lectin (AAEL010584-RF), F-spondin (AAEL007889-RB), Toll-pathway signaling NF-kappaB (AAEL007696-RF), sarm1 (AAEL014931-RD), and Class B Scavenger Receptor (AAEL011222-RA, AAEL000234-RA, AAEL000234-RL). The downregulated transcripts included trypsins (AAEL007818-RA, AAEL013713-RI), serine protease inhibitor (Serpin) (AAEL014141-RD, AAEL010769-RC), serine-type endopeptidase inhibitor activity (AAEL019468-RE), class B scavenger receptor (CD36 domain) (AAEL000234-RC, AAEL000234-RE, AAEL000234-RK, AAEL000234-RN), and the GPCR (AAEL002694-RB), amongst others.

### 2.5. Shared Transcripts between the In Vivo and the In Vitro Assays

**Figure 5:**
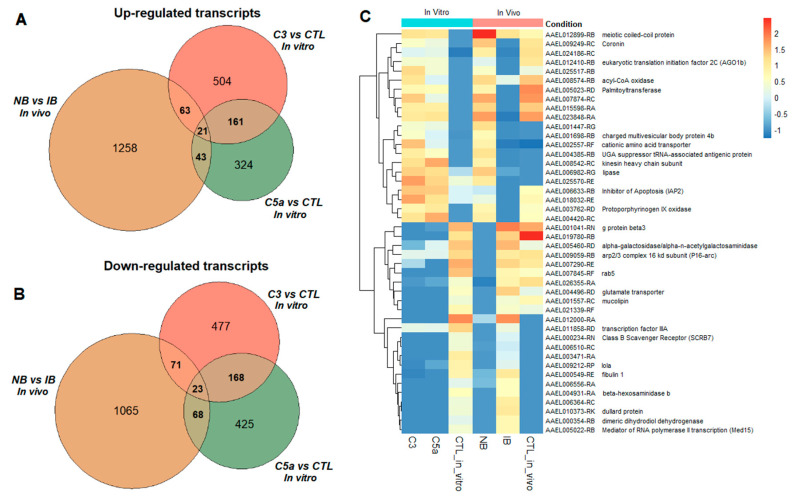
Differentially expressed protein-coding transcripts shared in response to both in vivo and in vitro experiments. Venn diagram of the up- (**A**) and downregulated (**B**) transcripts. The heatmap (**C**) shows the clustering of the 44 (21 up and 23 down) significantly regulated transcripts found in normal blood (NB), heat-inactivated blood (IB), and the in vitro experiments for treatments with hC3/hC5a and respective controls.

The comparison between transcripts found differentially expressed in live mosquitoes (in vivo assays) and those found in Aag2 cells (in vitro assays) shows 44 transcripts (21 up- and 23 downregulated) in common for both experiments (Figure 5, Table 1).

## 3. Discussion

As demonstrated in previous studies, our current study confirms that human blood ingestion induces significant mosquito transcriptome changes. Moreover, our study highlights how a blood meal containing active complement (NB group) leads to important changes in the transcriptome profile of *Ae. aegypti* females compared to those fed with sugar (SF group). A significant number of these differentially expressed genes are linked to digestive processes, maturation of eggs, suppression of the response to stimuli, and even activation of the immune response [28]. Since complement activation may be detrimental to the midgut tissue, *Ae. aegypti* females have developed a serine proteases mechanism to prevent complement-induced damage. Serine proteases cleave and inactivate human complement proteins and catalases that reduce the oxidative stress caused by the hemoglobin heme group, extending the time of mosquito survival by days [29]. Serine-type endopeptidases, such as trypsin, are essential for blood digestion and may be associated with DENV replication in the mosquito midgut [5]. Furthermore, it has been demonstrated that late-phase trypsin (5G1) is associated with a significant reduction in DENV2 replication in mosquito midgut [5]. Another study also showed that treating an infectious blood meal with a trypsin inhibitor reduced DENV-2 midgut titers and delayed viral dissemination [30]. Our study revealed that several serine-type endopeptidases upregulated are the most abundant transcripts after feeding with human red blood cells reconstituted with autologous plasma. In contrast, several serine-type protease inhibitors were found downregulated, which is consistent with previous studies suggesting that these enzymes are critical in blood meal digestion.

Since a blood meal has significant reproductive effects compared with a sugar meal (which is the primary source of energy), using whole abdomens instead of midguts allows evaluating broad transcriptome changes at the digestive and reproductive tracts level. Using all the abdominal tissues (ovaries, Malpighian tubules, fat body, and hemocytes), we obtained a comprehensive perspective on the effect of human complement over the mosquito physiology. Our comparison between SF and NB fed mosquitoes showed a higher overall number of upregulated transcripts than those found between the IB vs. SF groups. Our results suggest that the presence of the active complement induces a different type of response in the mosquitoes, especially when we see that more than 3000 transcripts are differentially expressed in both groups, but only half of those transcripts are shared between treatments.

The comparison between NB and IB fed mosquitoes showed that a significant number of upregulated genes in the NB group are associated with iron metabolism and oxidative stress. Previous studies have shown that these functions are important in defense against pathogens and protecting the arthropod against blood-derived factors, such as heme [31]. It is not surprising to find that transcripts related to iron metabolism were upregulated in NB fed mosquitoes since this is the way blood is up taken by mosquitoes. Regarding the oxidative stress in the midgut, previous studies suggest that the presence of heme significantly decreases reagent oxygen species as a protection mechanism induced by the heme overload product of hemoglobin digestion in the mosquito midgut [32]. Nevertheless, a recent study showed that the presence of heme in midgut induces significant changes in the expression of genes associated with energy metabolism and antioxidant activities, as is also shown in our study [31].

Heat-inactivation of plasma is mainly performed to denature complement proteins blocking the activation of all three complement pathways and avoiding complement-mediated signaling in adjacent cells. Other studies have demonstrated that heat treatment of serum reduces phagocytosis and chemotactic signaling in human immune cells [33]. Early research on the effect of temperature on serum reported the formation of heat-labile and heat-stable anticomplementary activity or ACA. The studies revealed that while heat-labile ACA can be completely inactivated through serum heating at 56 °C for 30 min, the heat-stable ACA increases progressively with continued heating [34,35], and it has been attributed to immunoglobulin aggregates [21]. In the current study, the transcripts highly represented as downregulated in the NB group compared to the IB inactivated fed mosquitoes were mainly associated with carbohydrate, lipid, and nitrogenous compounds metabolism, suggesting that heat inactivation of the plasma may decrease signals in these midgut-associated pathways. It is thought that feeding the mosquitoes with heat-inactivated plasma does not produce the same physiological signals as when the mosquitoes ingest an intact blood meal. Further studies are needed to investigate whether thermolabile ACA or other by-products of heat-inactivation induced the changes in transcripts observed in the present study.

One of the most noteworthy transcripts differences induced by active complement was the upregulation of AAEL009681-RB (Rhomboid-like protein) and AAEL029038-RA (Cecropin) with a fold increase higher than 5. Rhomboid proteins are associated with mitochondria homeostasis and cell signaling [36,37]. In the case of the AMP cecropin, approximately ten genes have been reported in *Ae. aegypti* mosquitoes [38], and different cecropins can be induced by the microbiota or the presence of pathogens [39,40,41]. Our results suggest that human complement plays a role in the modulation of cecropin transcription. A previous study using live *Ae. aegypti* mosquitoes (in vivo) suggested that activation of complement-related proteins in this species induce the production of AMPs, including cecropins [28]. Other in vivo studies also confirm the importance of Toll-pathway induced cecropins in controlling DENV replication in the mosquito *Ae. aegypti* [42,43]. Significant downregulation of cecropins upon infection with DENV [36] could represent a potential viral defense mechanism. Moreover, the increased expression of three defensins (AAEL003841-RB, AAEL027792-RA, and AAEL003857-RA) and three cecropins (AAEL029038-RA, AAEL029041-RA, and AAEL029047-RA) in the NB group compared to IB, may indicate that human complement proteins directly influence the immune response of *Ae. aegypti* and corroborate our previous findings suggesting that activation of human complement impacts the synthesis of AMPs [8].

We also aimed to evaluate the direct effect of two specific human complement proteins, hC3 and hC5a, on mosquito cells. First, hC3 protein is one of the most abundant human complement factors in plasma [44]. The hC3 can be physically adsorbed to tissue surfaces, and the binding to membranes in its fluid form as C3(H2O) may trigger responses in human tissue [45]. Thus, our current study suggests that in the event of C3 interaction with the midgut cells, it may also induce significant transcriptome changes. We observed more than 300 transcripts upregulated and more than 400 transcripts downregulated with an enrichment mainly in functions such as phosphotransferase and oxidoreductase activities. 

hC5a has shown an effect on DENV replication in our previous studies [8]. This molecule interacts with human cells through two possible receptors C5aR1 (C5a receptor 1 (CD88)) and C5aR2 (C5a receptor 2 (C5L2)). These receptors are mainly found in bone tissue and immune cells [46,47], where the association is associated with bone metabolism [48], induction of oxidative stress, and modulation of cytokine production in immune cells [49]. Our results show that transcripts modified by treatment with human hC5a are mainly associated with an increase in ion binding, phosphotransferase activity, and nucleotide-binding functions. We also observed decreased functions of actin-binding, cytoskeletal protein binding, GTPase regulator activity, and regulation of catalytic activity. Moreover, our results suggest that hC5a modified a larger number of transcripts than hC3, while only 329 transcripts were communally found modified in Aag2 cells after treatment with both complement proteins. In this regard, it is important to clarify that although both hC3 and hC5a were used in the in vitro experiments at the same concentration that was used in our previous study (1 μg/mL), the molarity in the solution was higher for hC5a (0.09 μM) than for hC3 (0.005 μM). A recent study showed that in patients with severe DENV infection, there is a dysregulation of the complement activity leading to a significant decrease in hC3 and an increase in the anaphylatoxins hC3a, hC4a, and hC5a [26]. Based on our previous studies and the shared IRT between the complement proteins, we hypothesize that mosquitoes feeding on DENV infected patients with severe clinical presentations (leading to higher complement activation) may present less infection and dissemination of the virus than those feeding on viremic asymptomatic individuals. In fact, previous studies suggest that asymptomatic DENV carriers may be more infectious to mosquitoes than those with symptoms at a given viremia level [27] that may be associated with the impact of human complement on the transcriptome of *Ae. aegypti* mosquitoes. Interestingly, we also observed that the commonly upregulated transcripts are associated with oxidoreductase activity and other enzymatic activities involved in mosquito metabolic processes, such as ion binding and phosphotransferase activity, suggesting a direct effect of human complement proteins in mosquito physiology.

**Figure 6:**
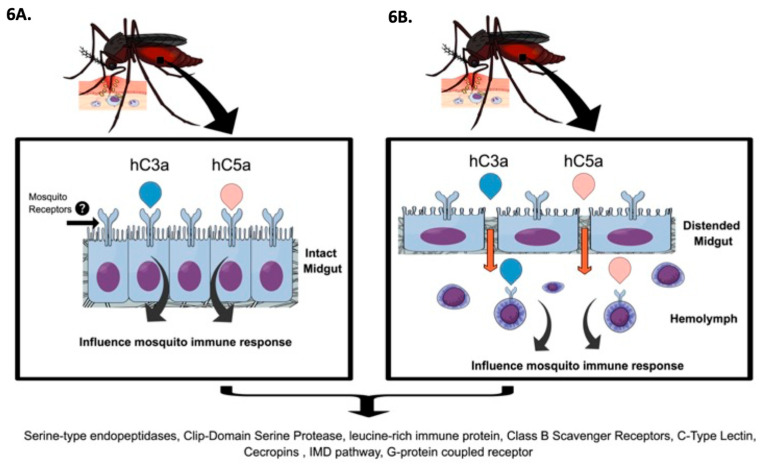
Graphical summary of our hypothesized mechanism of action of the effect of human complement proteins on mosquito cell transcripts. (**A**) Human complement proteins may interact with mosquito cells in the midgut; (**B**) Distention produced by the ingestion of blood may allow for the scape of complement molecules into the hemocoel where they could potentially interact with hemocytes.

Recent studies showed that the physical presence of blood (infected or uninfected) upon ingestion causes significant midgut distention. In the case of an infectious blood meal, this distention may allow the exit of viral particles into the hemolymph. Additionally, the expression of molecules involved in the degradation/remodeling of the midgut extracellular matrix during the blood-feeding may impact the scape of viruses [50,51]. We speculate that human complement proteins may interact with mosquito cells in two ways, through receptors in the surface of midgut cells before forming the peritrophic matrix (Figure 6A) or by escaping through the distended midgut cell layer during feeding (Figure 6B) where hC3 and hC5a molecules in the hemolymph could also interact with hemocytes. A previous study revealed that Aag2 cells are a hemocyte-like cell line and represent an appropriate in vitro model for the study of immune responses of *Ae. aegypti* mosquitoes [52], whereby we used it to evaluate the potential effect of complement proteins on mosquito immune responses. We believe that these interactions are responsible for the transcript changes observed in this study. Although no specific receptors for any human complement proteins have been identified in mosquito cells so far, these molecules may start transcription changes by binding proteins on the mosquito cells surface or by internalization via pores or endocytosis. In humans, clathrin-dependent endocytosis of the GPCR receptor is activated upon interaction with hC5a [53] and internalization of hC5a and leads to inflammation and oxidative burst [54]. Other studies suggest that hC3 can be internalized via an unknown mechanism, and its internal cleavage and production of hC3a leads to inflammatory responses [55,56]. Binding of hC3 can direct the intracellular route of the cargo modulating immune responses [57]. Mosquitoes may have evolved different routes to dispose of harmful components in the human blood (as noted above) in addition to a mechanism in which the human blood can be an immune buster protecting the arthropod from harmful pathogens.

The current study demonstrates the effect of human complement proteins on mosquito physiology; however, we acknowledge that it has limitations. First, we only measured transcripts at one point in time for both experiments, at 3 h for the in vivo, and at 24 h for the in vitro experiments. In addition, the in vivo experiment was only performed in duplicates. We are planning to expand these experiments and measure additional time points in the in vivo experiments (6, 12, 24, and 48 h) in addition to compare transcripts in DENV infected mosquitoes vs. those in non-infected to determine changes in the transcripts in relation to infection status. We believe that the findings of the current study support previous studies demonstrating the impact of human blood components in the physiology and immune response of arthropod vectors and highlights the importance of using normal non-inactivated human blood component to accurately evaluate vector competence. Furthermore, *Ae. aegypti* has a large number of transcripts involved in immune system processes; however, there is currently no curated list of these transcripts, to the best of our knowledge. Therefore, the proposed list established in this work of IRTs can serve as a frame of reference to establish a complete and refined list of immune-related genes of *Ae. aegypti* that can be used by the research community. In conclusion, our study shows that active complement induces significant changes in the transcriptome of *Ae. aegypti* mosquitoes with an important number of modulated genes involved in immune responses.

## 4. Materials and Methods 

### 4.1. Blood Source

The Institutional Review Board (IRB) approval for collecting human blood from healthy volunteers was granted by the University of South Carolina IRB (IRB# Pro00045351, approval date: 25 August 2015 Human whole peripheral blood was collected in ethylenediaminetetraacetic acid (EDTA) and processed for use in the experiments. Briefly, plasma was separated from whole human blood by centrifugation, and the red blood cells (RBCs) were washed three times in 1 × PBS and kept at 4 °C until use. For the in vivo experiments, the inactivation of plasma was accomplished by heating the plasma for 30 min at 56 °C. Inactivation of all complement pathways was verified using the Complement System Screen kit (Euro Diagnostica, Malmo, Sweden) according to the manufacturer’s instructions.

### 4.2. Maintenance of Ae. aegypti Adult Mosquitoes and Cell Line

*Ae. aegypti* mosquitoes (Rockefeller strain, BEI Resources, Atlanta, USA) were reared at 27 ± 1 °C, 75 ± 5% RH, with a photoperiod of 16:8 h (L:D). Adults were maintained on 10% sucrose *ad libitum*. Female mosquitoes (*n* = 60) 5 to 10 days-old were feed with a mixture of human RBC’s in a 1:1 ratio with either heat-inactivated or normal non-inactivated autologous plasma (Londono-Renteria et al., 2016). Aag2 *Ae. aegypti* cells, originating from embryonic cells [58], were grown at 28 °C, Schneider *Drosophila* media supplemented with 10% heat-inactivated fetal bovine serum (Gemini, CA, USA), 1% penicillin–streptomycin, and 1% tryptose phosphate broth (Complete media) (Sigma, MO, USA).

### 4.3. Mosquito Blood-Feeding

To measure the in vivo effect of human complement inactivation on mosquito physiology, female adult mosquitoes were fed with either heat-inactivated blood plasma (500 μL inactivated plasma + 500 μL of homologous packed RBC), or normal non-inactivated plasma (500 μL normal plasma + 500 μL homologous packed RBC). Mosquitoes were fed for 30 min at room temperature using 1 mL of blood mixture in a Hemotek feeder maintained at 37 °C. Engorged females were sorted in different cages and held under standard conditions. Age-matched sugar-fed mosquitoes were used as controls. Three hours (3 h) post-feeding, whole abdomens were dissected and transferred to 1.5-mL tubes in pools of 10 abdomens. The tissue was homogenized in RLT buffer (Qiagen, CA, USA) with Basal Medium Eagle (BME, Thermo Scientific, Waltham, MA, USA) as the lysis buffer.

### 4.4. Aag2 Cells Exposure to Human Complement Protein hC3 and hC5a

To measure the in vitro effect of the specific human complement proteins C5a and C3, we used the Aag2 *Ae. aegypti* cell line. Cells were seeded in a 24-well cell culture plate 24 h before the experiment. Cells were treated with 1 μg/mL of either recombinant hC3 (Abcam) or hC5a (R&D systems) dissolved in complete media [8]. Control cells contained complete media only. After 24 h incubation at 28 °C, cells were harvested using the lysis buffer described above.

### 4.5. Mosquito RNA Isolation and Sequencing

Mosquito RNA was extracted using a Quick-RNA miniprep kit (Zymo Research, Irvine, CA, USA). Each in vivo experiment was conducted in duplicates for the feedings with sugar (SF), heat-inactivated (IB) blood, and normal blood (NB) (*n* = 6). Each in vitro experiment was conducted in triplicates for Aag2 control untreated cells, cells exposed to hC3, and cells exposed to hC5a (*n* = 9), for a total of 15 samples sequenced. A total of 3 ng of RNA per sample was sent for sequencing to LC Sciences (Houston, TX, USA), where the sample QC, library preparation (with enrichment for mRNA using poly(A) selection, single-molecule clonal amplification), and Illumina sequencing (San Diego, CA, USA) was performed.

### 4.6. RNA-Seq Differential Expression Analysis

Raw single reads were subjected to sequence quality control using FastQC v0.11.9 (http://www.bioinformatics.babraham.ac.uk/projects/fastqc/) and were quality filtered using Fastp v0.20.0 (Chen et al., 2018) to eliminate low quality and short reads (<10 pb). High quality processed reads were mapped to the *Ae. aegypti* genome assembly AaegL5.2 [59] (https://www.vectorbase.org/) using STAR v2.7 [60] and Hisat2 v2.20 [61]. Mapped reads were counted using RNA-Seq by Expectation-Maximization (RSEM) v1.3.3 [62].

The package NOIseq v2.31.0 [63] was used to perform differential expression analysis in the R software environment (https://www.r-project.org/), implementing the non-parametric approach NOIseqBio that improves the handling of biological variability specific to each gene, and is very successful in controlling the high false discovery rate (FDR) in experiments with biological replicates. The count filter was used to remove transcripts with Counts per Million (CPM) <1 in the samples, which avoids noise from lowly expressed transcripts. The normalization method used was the Trimmed Mean of M-values (TMM) approach. To identify those differentially expressed (DE) between comparisons, the q-value cut-off 0.95 was implemented, and only protein-coding transcripts with a fold change ≥4 (Log2FC ≥2) were analyzed.

The gene description and gene ontology (GO) terms of DE transcripts were obtained from VectorBase.org using the BiomaRt package v.2.44.1 [64,65]. The Gene Set Enrichment Analysis (GSEA) was performed using g: Profiler (https://biit.cs.ut.ee/gprofiler/gost). The transcripts related to immune response (IRTs), which include immunity and other processes, such as small regulatory RNAs, apoptosis, and autophagy, were identified considering the list of 477 transcripts obtained by in silico comparative genomic analyses and manual annotation that have established or putative associations with defense mechanisms published by Bonizzoni et al. [66]. Because this list of transcripts was generated with an older genome assembly (AaegL1.2), only 243 of the 477 transcripts were found in the current assembly (AaegL5.2). With VectorBase.org, it was possible to convert some of the old IDs to the new ones; others have been lost. To look for new IRTs in the current assembly, the following functional annotations were used: GO:0002376 (immune system process), GO:0006955 (immune response), GO:0045087 (innate immune response), GO:0006959 (humoral immune response), GO:0006952 (defense response), GO:0006915 (apoptotic process), GO:0042981 (regulation of apoptotic process), GO:0008592 (regulation of Toll signaling pathway), GO:0008236 (serine-type peptidase activity), GO:0004252 (serine-type endopeptidase activity), GO:0004867 (serine-type endopeptidase inhibitor activity), GO:0008234 (cysteine-type peptidase activity), and GO:0004869 (cysteine-type endopeptidase inhibitor activity). The updated and curated list of IRTs contains 883 transcripts (Table S3).

The GSEA plots were generated using the ggplot2 package v3.3.0 [67,68]. The heatmaps, clustered on expression profiles, were created to visualize changes in the experiment’s profiles between transcripts. The z-score, shown as the scale key on each figure’s side, represents the normalized gene expression measurements, where 0 has no difference from the mean. To illustrate the transcripts’ expression, red color represents positive fold change (FC), indicating higher expression in the corresponding transcripts, and blue color represents negative FC, indicating decreased expression. The heatmaps were generated using the Pheatmap package v1.0.12 (https://cran.r-project.org/web/packages/pheatmap/index.html). The Venn diagrams represented the number of differentially expressed transcripts when comparing the different treatments (FC ≥ 4 and q-value > 0.95) and were generated using the Venneuler package v1.1-0 (http://www.rforge.net/venneuler/index.html).

## Figures and Tables

**Table 1:** Forty-four shared differentially expressed protein-coding transcripts in response to treatment with human complement both in vivo and in vitro. The top of the table shows the upregulated (positive fold change (FC) values), and the bottom of the table shows the downregulated (negative FC) transcripts. Ctl denotes Aag2 untreated control cells.

Transcript_ID	Gene Description (Gene Ontology Molecular Function and Description)	Log2FC		
		hC3/Ctl	hC5a/Ctl	NB/IB
AAEL001698-RB	*Charged multivesicular body protein 4b* (GO:0007034, vacuolar transport)	2.80	2.06	5.46
AAEL007290-RE	*None* (None)	2.93	2.90	3.06
AAEL001447-RG	*None*	3.40	4.26	3.91
AAEL002557-RF	*Cationic amino acid transporter (GO:0022857, transmembrane transporter activity)*	3.41	2.91	5.27
AAEL005460-RD	*alpha-galactosidase/alpha-n-acetylgalactosaminidase (GO:0003824;GO:0016787;GO:0016798;GO:0004553, catalytic activity; hydrolase activity; hydrolase activity, acting on glycosyl bonds)*	3.78	3.75	3.54
AAEL025517-RB	*None (GO:0005524;GO:0004672, ATP binding; protein kinase activity)*	3.79	3.47	5.92
AAEL012899-RB	*Meiotic coiled-coil protein, putative GO:0003690, double-stranded DNA binding)*	3.96	3.58	6.30
AAEL026355-RA	None (GO:0005515, protein binding)	4.25	3.23	4.85
AAEL008574-RB	*Acyl-CoA oxidase (GO:0071949;GO:0003997;GO:0050660;GO:0016627, FAD binding; acyl-CoA oxidase activity; flavin adenine dinucleotide binding)*	4.45	4.36	5.09
AAEL009249-RC	*Coronin (GO:0005515, protein binding)*	4.58	5.13	4.97
AAEL009212-RP	*Lola (GO:0005515, protein binding)*	4.86	4.88	6.08
AAEL007845-RF	*Rab5 (GO:0005525;GO:0003924, GTP binding; GTPase activity)*	4.88	5.16	6.89
AAEL001557-RC	*Mucolipin (GO:0005261, cation channel activity)*	4.89	2.70	5.76
AAEL005022-RB	*Mediator of RNA polymerase II transcription subunit 15 (Med15) (GO:0003712, transcription coregulator activity)*	5.01	5.26	8.12
AAEL003762-RD	*Protoporphyrinogen IX oxidase (GO:0016491;GO:0004729, oxidoreductase activity; oxygen-dependent protoporphyrinogen oxidase activity)*	5.15	6.23	3.13
AAEL015598-RA	*None (GO:0016787, hydrolase activity)*	5.21	2.97	2.24
AAEL019780-RB	*None (GO:0008410, CoA-transferase activity)*	5.27	5.19	2.62
AAEL005023-RD	*Palmitoyltransferase (None)*	5.33	6.30	5.14
AAEL021339-RF	*None (None)*	5.50	3.79	3.93
AAEL004931-RA	*Beta-hexosaminidase b (GO:0102148;GO:0004563;GO:0016787;GO:0016798;GO:0004553, N-acetyl-beta-D-galactosaminidase activity; beta-N-acetylhexosaminidase activity)*	5.67	3.41	6.73
AAEL024186-RC	*None (GO:0003779;GO:0030276;GO:0005543, actin binding; clathrin binding; phospholipid binding)*	5.87	3.00	6.26
AAEL006633-RB	*Inhibitor of Apoptosis (IAP) (None)*	−7.22	−7.08	−4.47
AAEL006510-RC	*None (GO:0003723;GO:0008190, RNA binding; eukaryotic initiation factor 4E binding)*	−6.61	−6.04	−6.91
AAEL012000-RA	*None (None)*	−6.42	−6.29	−4.70
AAEL009059-RB	*Arp2/3 complex 16 kd subunit (P16-arc) (None)*	−5.86	−5.74	−5.88
AAEL001041-RN	*Guanine nucleotide-binding protein beta 3 (g protein beta3) (GO:0005515, protein binding)*	−5.72	−5.00	−4.14
AAEL000234-RN	*Class B Scavenger Receptor (CD36 domain) (None)*	−5.50	−3.75	−3.05
AAEL012410-RB	*Eukaryotic translation initiation factor 2C (GO:0003676;GO:0005515, nucleic acid binding; protein binding)*	−5.50	−4.20	−3.32
AAEL008542-RC	*Kinesin heavy chain subunit (GO:0005524;GO:0008017;GO:0003777, ATP binding; microtubule binding; microtubule motor activity)*	−5.04	−4.91	−4.09
AAEL023848-RA	*None (None)*	−4.87	−2.81	−6.23
AAEL007874-RC	*None (GO:0047372, acylglycerol lipase activity)*	−4.76	−4.63	−4.37
AAEL004496-RD	*Glutamate transporter (GO:0015293, symporter activity)*	−4.66	−4.52	−5.88
AAEL010373-RK	*Dullard protein (GO:0016791, phosphatase activity)*	−4.29	−4.16	−4.68
AAEL006364-RC	*None (GO:0045127, N-acetylglucosamine kinase activity)*	−4.24	−4.11	−7.08
AAEL004420-RC	*None (None)*	−4.19	−4.06	−5.29
AAEL011858-RD	*Transcription factor IIIA (GO:0003676;GO:0008270, nucleic acid binding; zinc ion binding)*	−4.19	−4.07	−3.54
AAEL006982-RG	*Lipase (GO:0052689, carboxylic ester hydrolase activity)*	−4.18	−4.07	−2.50
AAEL025570-RE	*None (GO:0005524;GO:0003774;GO:0005515, ATP binding; motor activity; protein binding)*	−4.12	−6.57	−2.59
AAEL004385-RB	*UGA suppressor tRNA-associated antigenic protein (GO:0003824;GO:0016740;GO:0016785, catalytic activity; transferase activity; transferase activity)*	−4.11	−3.98	−5.09
AAEL018032-RE	*None (None)*	−3.97	−3.43	−6.07
AAEL000549-RE	*Fibulin 1 (GO:0005509;GO:0005515, calcium ion binding; protein binding)*	−3.75	−3.62	−4.69
AAEL000354-RB	*Dimeric dihydrodiol dehydrogenase (GO:0016491, oxidoreductase activity)*	−3.69	−3.56	−3.09
AAEL006556-RA	*None (None)*	−2.47	−2.43	−5.21
AAEL003471-RA	*None (GO:0003676;GO:0008270, nucleic acid binding; zinc ion binding)*	−2.44	−2.40	−3.73

## Supplementary Materials

The following are available online at https://www.mdpi.com/1422-0067/21/18/6584/s1.

## Abbreviations

YFV	Yellow fever virus
DENV	Dengue virus
CHIKV	Chikungunya virus
ZIKV	Zika virus
MAC	Membrane attack complex
hC3	Human complement protein C3
hC5a	Human complement protein C5a
AaMCR	*Ae. aegypti* macroglobulin complement-related proteins
AMP	Antimicrobial peptides
TBV	Transmission blocking vaccines
LVP_AGWG	Liverpool Aedes Genome Working Group
STAR	Spliced Transcripts Alignment to a Reference
RSEM	RNA-Seq by Expectation-Maximization
FDR	False discovery rate
CPM	Counts per Million
TMM	Trimmed Mean of M-values
DE	Differentially expressed
IB	Blood with heat-inactivated plasma
NB	Blood with normal plasma
SF	Sugar-fed
FC	Fold change
GSEA	Gene Set Enrichment Analysis
MF	Molecular functions
GO	Gene Ontology
IRT	Immune response transcripts
5G1	Late-phase trypsin
ACA	Anticomplementary activity
C5aR1	C5a receptor 1 (CD88)
C5aR2	C5a receptor 2 (C5L2)
GTP	Guanosine-5’-triphosphate
EDTA	Ethylenediaminetetraacetic acid
RBC	Red blood cells
PBS	Phosphate Buffered Saline
BME	Basal Medium Eagle
KOAPF	K-State Open Access Publishing Fund
